# What pops out in positional priming of pop-out: insights from event-related EEG lateralizations

**DOI:** 10.3389/fpsyg.2014.00688

**Published:** 2014-07-02

**Authors:** Ahu Gokce, Thomas Geyer, Kathrin Finke, Hermann J. Müller, Thomas Töllner

**Affiliations:** ^1^Department of Psychology, Ludwig-Maximilians-UniversitätMünchen, Germany; ^2^School of Psychological Sciences, Birkbeck, University of LondonLondon, UK; ^3^Graduate School of Systemic Neurosciences, Ludwig-Maximilians-Universität MünchenMünchen, Germany

**Keywords:** attention, visual search, sequence effects, PCN, CDA, LRP, Ppc

## Abstract

It is well established that, in visual pop-out search, reaction time (RT) performance is influenced by cross-trial repetitions versus changes of target-defining attributes. One instance of this is referred to as “positional priming of pop-out” (pPoP; Maljkovic and Nakayama, 1996). In positional PoP paradigms, the processing of the current target is examined depending on whether it occurs at the previous target or a previous distractor location, relative to a previously empty location (“neutral” baseline), permitting target facilitation and distractor inhibition to be dissociated. The present study combined RT measures with specific sensory- and motor-driven event-related lateralizations to track the time course of four distinct processing levels as a function of the target’s position across consecutive trials. The results showed that, relative to targets at previous target and “neutral” locations, the appearance of a target at a previous distractor location was associated with a delayed build-up of the posterior contralateral negativity wave, indicating that distractor positions are suppressed at early stages of visual processing. By contrast, presentation of a target at a previous target, relative to “neutral” and distractor locations, modulated the elicitation of the subsequent stimulus-locked lateralized readiness potential wave, indicating that post-selective response selection is facilitated if the target occurred at the same position as on the previous trial. Overall, the results of present study provide electrophysiological evidence for the idea that target location priming (RT benefits) does not originate from an enhanced coding of target saliency at repeated (target) locations; instead, they arise (near-) exclusively from processing levels subsequent to focal-attentional target selection.

## INTRODUCTION

### INTER-TRIAL PRIMING EFFECTS

Visual search performance on a given trial is determined not only by the currently active top-down and bottom-up biases (e.g., Corbetta and Shulman, 2002), but also through sensory and motor-related events that occurred on the previous trial(s) – a class of memory effects commonly referred to as “inter-trial priming.” Over the last two decades, a number of target attributes have been revealed to selectively, or interactively, contribute to intertrial priming, including stimulus positions (e.g., Maljkovic and Nakayama, 1996; Geyer et al., 2007), stimulus features (e.g., Maljkovic and Nakayama, 1994; Kristjánsson and Driver, 2008), visual dimensions (e.g., Müller et al., 1995; Found and Müller, 1996), sensory modalities (e.g., Spence et al., 2001; Töllner et al., 2009), objects (e.g., Kristjánsson et al., 2008), motor responses (e.g., Töllner et al., 2008), as well as components of the task set adopted by participants to optimize performance (e.g., Rangelov et al., 2011, 2013). The general finding is that repetitions relative to changes of target-defining attributes leads to speeded visual search performance – an effect putatively attributed to a combination of facilitation of previous target, and inhibition of previous distractor, attributes (e.g., Maljkovic and Nakayama, 1996; Kristjánsson and Driver, 2008; Lamy et al., 2008). However, previous studies have yielded mixed accounts with regard to the mechanisms underlying inter-trial priming effects. Also, to our knowledge, no study has as yet systematically examined the locus of *positional priming of pop-out* (pPoP; e.g., Maljkovic and Nakayama, 1996; for recent studies see, e.g., Geyer et al., 2010; Gokce et al., 2013).

### PRE-ATTENTIVE VERSUS POST-SELECTIVE ORIGINS OF INTER-TRIAL PRIMING EFFECTS

This locus-of-effect debate centers mainly on the level of representation that is primed by the repeated targets. According to the “pre-attentive” view, inter-trial priming facilitates early sensory processes, such as the selection of the target by focal attention (e.g., Maljkovic and Nakayama, 2000; Goolsby and Suzuki, 2001; Müller et al., 2003, 2010; Wolfe et al., 2003; Meeter and Olivers, 2014). The “post-selective” view, by contrast, assumes that inter-trial priming facilitates processes after target selection, such as processes of response selection (Cohen and Magen, 1999; Mortier et al., 2005; Theeuwes et al., 2006). The available evidence suggests, however, that these accounts are not mutually exclusive. For instance, Töllner et al. (2008) investigated the locus of dimension and response priming effects in visual search by coupling mental chronometry to event-related lateralizations (ERLs). They found that repetitions versus changes of the target-defining dimension selectively modulated a sensory-driven ERL [the posterior contralateral negativity (PCN) wave], whereas repetitions versus changes of the target’s response-defining attribute selectively modulated a motor-driven ERL [the response-locked lateralized readiness potential (rLRP) wave]. This dissociation suggests that perceptual and response priming are coexisting phenomena. Closely in line with this ERL pattern, Lamy et al. (2010) proposed a “dual-stage” account of inter-trial priming, in which both early and late priming effects can co-occur, but differ with regard to their temporal characteristics: perceptual priming builds up rapidly (i.e., within 100–300 msec) upon the onset of the trial display. Response priming, by contrast, becomes manifest only later during the trial, at around 400 msec after stimulus onset.

The present study was designed to test one core assumption relating to accounts of *positional PoP*, namely, that the memory trace underlying positional priming effects enables more efficient – that is, faster – visual selection (e.g., Maljkovic and Nakayama, 1994, 1996, 2000; see also McPeek et al., 1999). The specific questions addressed are whether positional priming facilitates focal-attentional selection of the target and, if so, whether there are differences between the memory traces underlying target and distractor location priming (see, e.g., Geyer et al., 2007, 2010; Finke et al., 2009, for experimental and neuropsychological evidence in favor of distinct mechanisms mediating target and distractor location priming). While the studies reviewed above support a role of feature priming mechanisms in attentional guidance, it remains an open issue whether positional priming acts on attention-guiding representations as well.

Regarding the locus of positional PoP, Maljkovic and Nakayama (1996, 2000) proposed that positional priming facilitates focal-attentional target selection. One way of how this may be implemented in the human vision system is that pre-attentive saliency computations are speeded for previous target locations, and slowed for previous distractor location. As a consequence, visual selection is faster for targets occurring at previous target locations, and slower for targets at previous distractor locations. In a sense, target facilitation and distractor inhibition could be considered as instances of a spatial weighting mechanism, increasing or, respectively, decreasing priority signals at the level of the attention-guiding master map. Note, tough, that Maljkovic and Nakayama’s (1996) conclusions were based solely on behavioral measures. In fact, there are only few studies that have examined the locus of positional priming at the neural level. For example, using functional magnetic resonance imaging (fMRI), Geng et al. (2006; see also Kristjánsson et al., 2007; Rorden et al., 2011) found repeated relative to changed target positions leading to repetition suppression effects in a variety of attentional control areas, including the intra-parietal sulcus (IPS) and the frontal eye fields (FEF). However, there are two limitations associated with these studies: The first concerns the temporally sluggish nature of the blood-oxygen-level dependent (BOLD) signal, which makes inferences about the timing of positional PoP effects impossible. Second, the above-mentioned studies are unable to dissociate target location priming from distractor location priming. Kristjánsson et al. (2007) and Rorden et al. (2011), for example, compared variations of the BOLD signal between same- and different-location trials, where, in the latter, the target appeared always at a previous distractor location. In other words, there was no “neutral” baseline condition against which the effects of target presentation at previous target and, respectively, distractor locations could be compared. Thus, one cannot tell whether the positional inter-trial effects observed by Kristjánsson et al. (2007) (i.e., reduced neural activity for same-vs. different-location trials) reflect facilitation for previous target locations and/or inhibition for previous distractor locations.

### RATIONALE OF THE PRESENT STUDY

On this background, we recorded the electroencephalogram in the current study to track the time-course of pop-out signal processing on a millisecond-by-millisecond basis. To adequately asses the—pre-attentive vs. post-selective—locus, or loci, of positional priming effects, we focused our analyses on a number of particular ERLs that can be linked directly to pure perceptual and pure motor processes, respectively. Similar to the study design devised by Maljkovic and Nakayama (1996), these ERLs were recorded while participants performed a visual pop-out search task, in which the response was based—independent of the target-defining color—on the location of the cut-off section or “notch” (top vs. bottom) of the target stimulus. This compound task required participants to first select the unique-color target from the distractors, before they could extract the notch position required to decide upon the correct motor response. Furthermore, the design of the present study included a neutral baseline condition – in which the current target was presented at a previously empty location – permitting effects of re-presentation of the target at the same (i.e., the previous target) location versus presentation of the target at a previous distractor location to be dissociated.

The first component of interest was a negative waveform elicited ∼175–300 msec post-stimulus over the visual areas contralateral to the attended target stimulus. This PCN (also called N2-posterior-contralateral), which is generated in the ventral occipito-temporal cortex (see, e.g., Hopf et al., 2002), is widely accepted to reflect the deployment of focal attention in visual space (e.g., Luck and Hillyard, 1994; Eimer, 1996; Woodman and Luck, 1999; Hickey et al., 2006, 2009; McDonald et al., 2009; Töllner et al., 2012a). Of note, modulations of the PCN have been documented already for different types of non-spatial priming, including feature (Eimer et al., 2010) and dimension priming (Töllner et al., 2008). For instance, Eimer et al. (2010) recently reported that the elicitation of the PCN depended on whether there was a change of the target- and distractor-defining colors across successive trials: the PCN was speeded and enhanced for cross-trial repetitions relative to changes of the target- and distractor-defining colors, which has been taken to indicate that featural priming enables more efficient target selection^1^.

The next component of interest was a second negativity that is likewise elicited over the visual areas contralateral to the attended hemifield, however, at later latencies, starting from ∼350 msec post-stimulus. This contralateral delay activity (CDA, or sustained-posterior-contralateral-negativity) has originally been observed in working-memory (WM) studies and is assumed to reflect the active maintenance of information in WM (e.g., Vogel and Machizawa, 2004; Wiegand et al., 2013b). Recent studies, however, identified this ERL also in visual search tasks, if participants had to extract detailed object identity information from visual WM to solve the task (see Klaver et al., 1999; Mazza et al., 2007; Jolicæur et al., 2008). For instance, Töllner et al. (2013) recently showed that CDA amplitudes scale with task difficulty: CDA waves were increased in amplitude when it was more difficult for observers to extract the target’s exact featural identity from WM. Accordingly, Töllner et al. (2013) suggested that the CDA does reflect not only maintenance of, but also access to, detailed object information in WM.

Lastly, we concentrated on the LRP as an online marker for response-related processes in the present paradigm (e.g., Coles, 1989; Eimer and Coles, 2003). Depending on how this ERL is extracted from the event-related potential (ERP), the time demands of two distinct processing stages can be inferred. When computed relative to the onset of the search display [i.e., stimulus-locked LRP (sLRP)], the timing of the LRP indicates the time it takes for observers to select the appropriate response in accordance with a pre-established task set (specifying the stimulus-response mapping). When computed relative to the onset of the motor response [i.e., response-locked LRP (rLRP)], the timing of the LRP indicates the time it takes for observers to produce the actual response (see Töllner et al., 2012b, for further details).

Following Maljkovic and Nakayama (1996), three hypotheses can be made regarding an early locus of position priming effects: repeating target, but not distractor, positions may facilitate focal-attentional selection of the target. Alternatively, targets presented at previous distractor, but not those at previous target, locations slow attentional selection. Third, it is also possible that both target presentation at the former target location and target presentation at a former distractor location influence the speed of visual target selection.

## MATERIALS AND METHODS

### PARTICIPANTS

Fourteen observers (female: 8, mean age: 23, SD: 1.74 years), recruited from the participant panel of the unit of Experimental Psychology, LMU Munich, took part in the study. All participants had normal or corrected-to-normal visual acuity, and all reported normal color vision and being right-handed. Participants were naïve as to the purpose of the study. Informed consent was obtained prior to the start of the experiment and anonymity of observers’ data was guaranteed. Participants were paid at a rate of 8 € (∼10 USD) per hour, or received course credits for their participation.

### APPARATUS AND STIMULI

The search display was composed of four stimuli presented on a gray background (20 cd/m^2^): always one target presented amongst three distractor diamonds (stimulus size: 1.51° × 1.51° of visual angle). The target and distractors were either red or green (equiluminant colors: 28 cd/m^2^), and they were arranged equidistantly around a virtual circle (7.57° in diameter), with a black central fixation cross (size: 0.76° × 0.76° size; luminance: 0.3 cd/m^2^). All stimuli had a cut-off section (or “notch”; size: 0.25°) at either the top or the bottom part. Participant’s task was to indicate the position of the target notch (top vs. bottom) by pressing the corresponding mouse button. In the first experimental session, observers with odd/even participant numbers responded to the top notch with their left/right, and to the bottom notch with the right/left, thumb. These mappings were reversed in the second session. A standard PC with Microsoft Windows XP Prof operating system controlled stimulus presentation and response recording. The experimental control software was purpose-written in C++. Stimuli were presented on a 19-inch CRT screen (AOC; Amsterdam, Netherlands), with screen resolution set to 1024 × 768 pixels a refresh rate of 85 Hz. Participants viewed the screen from a distance of ∼75 cm. The experimental cabin was sound attenuated, dimly lighted, and electrically shielded.

### PROCEDURE

**FIGURE 1:**
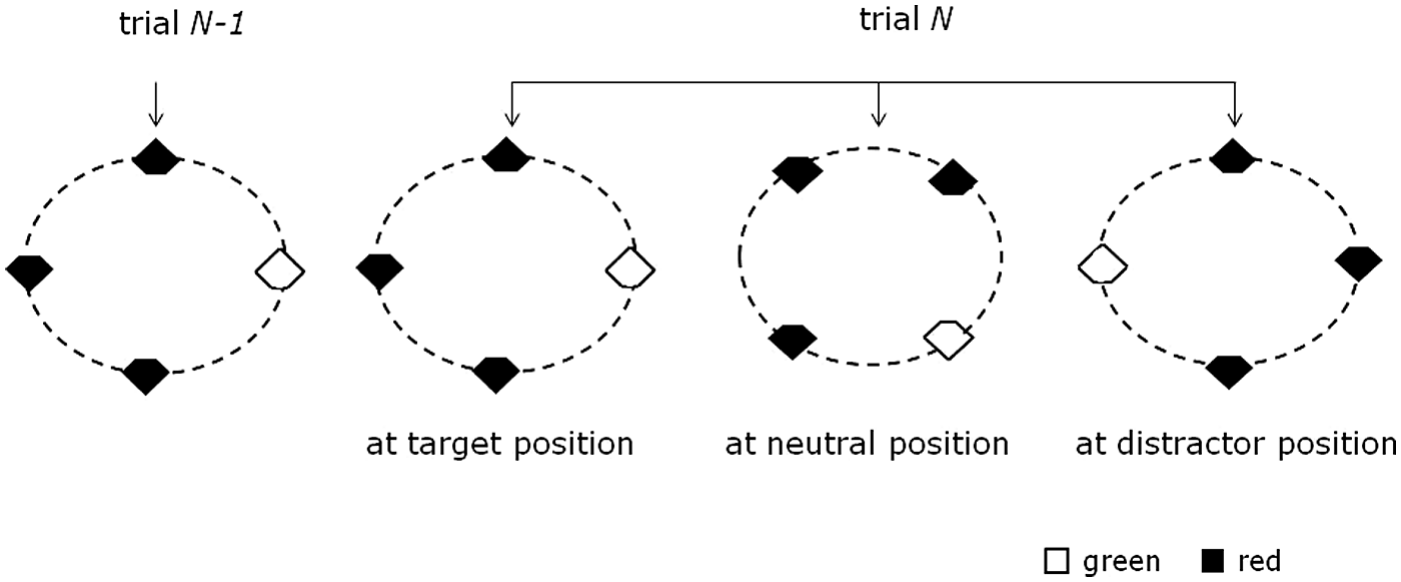
**Schematic of cross-trial target location transitions in the present study.** In a given display, four out of eight locations were occupied by the search items (one target, three distractors). The four items could form either a virtual square or diamond configuration. The target was equally likely to appear at any of the four corners of the square or diamond configuration. (The near-circular ellipses were not presented in the actual experiment; they are presented here for illustration purposes only.)

The experiment consisted of two consecutive sessions, each comprising eight blocks of 112 trials, yielding a total number of 1792 trials. At the beginning of the experiment, observers practiced the experimental task in a block of 32 trials (data not recorded). On a given trial, the fixation cross was presented for 500 msec, which was followed by the stimulus display presented for 200 msec (see **Figure 1**). The trial was terminated by the observer’s response. When observers responded too slowly (i.e., trial RT > 1 s) or incorrectly, they received immediate error feedback on the screen (i.e., “Too slow”/“Error”), for 1 s. The inter-trial interval was jittered, ranging randomly between 0.95 and 1.05 s. Observers were instructed to maintain gaze at the central fixation cross and to respond as fast and as accurately as possible. At the end of every fourth block, observers took a short break. Mean RTs and error rates were displayed to the observers at the end of each block.

### STUDY DESIGN

The search display consisted of eight possible stimulus locations arranged on a circular layout. On a given trial, however, only four out of the eight locations were occupied (i.e., one target, three distractors). The four items could form either a virtual square or diamond configuration. The target was equally likely to appear at any of the four corners of the square or diamond configuration. Note that, since the primary aim of the study was to examine lateralized ERP waves, for the diamond configuration, only trials with targets presented at lateral locations were included in the data analyses. In half of the trials, the target was red and the distractors green, and *vice versa* in the other half. The cut-off section of each stimulus (top vs. bottom notch) was determined randomly on each trial. With regard to the previous trial n-1, the target on the current trial n could appear at one of three possible locations: at a previous target location (*TT*; 33% of all trials), at a previous distractor location (*TD*; 33% of trials), or a previously empty, that is, “neutral” location (*TN*; 33% of trials).

### EEG RECORDING AND DATA ANALYSIS

The EEG was recorded from 64 Ag/AgCl electrodes with a sampling rate of 1 KHz. The electrodes were placed according to the international 10/10 system (American Electroencephalographic Society, 1994). EEG signals were amplified by BrainAmp DC amplifiers (Brain Products, Munich, Germany) using a 0.1- to 250-Hz band-pass filter. Electrophysiological signals were filtered oﬄine with a 0.1–40-Hz band-pass (Butterworth 0 phase, 24 dB/Oct). All electrodes were referenced to FCz during recording and re-referenced oﬄine to averaged mastoids. Impedances were kept below 5 kΩ. An infomax independent-component analysis was conducted to identify and backtransform blink and/or horizontal eye movement artifacts. Only trials with correct responses were included in the analysis. Before averaging, signals exceeding ±60 μV and lower than 0.5 μV (indicating “dead” channels) were removed from the analysis on an individual-channel basis. For the PCN and CDA analyses, the EEG data were epoched into 500-msec periods relative to a 200-msec pre-stimulus baseline, which was used for baseline correction. In order to isolate lateralized PCN and CDA difference waves from the non-lateralized ERPs, the waveforms at the electrodes PO7/8 ipsilateral to the side of the target location were subtracted from the contralateral ERPs. The PCN and CDA latencies were defined as the maximum negative deflection within the time windows 150–350 msec and, respectively, 350–500 msec post-stimulus. PCN and CDA amplitudes were determined by averaging five sample points before and after the respective maximum deflections. For the LRP analyses, we extracted both stimulus-locked and response-locked LRPs. The response-locked LRPs were obtained by epoching the EEGs into 1-sec periods (800 msec before and 200 msec after the response onset). The stimulus-locked LRPs were obtained by epoching the EEGs into 800-msec periods relative to a 200-msec pre-stimulus baseline. In order to extract the LRPs from the non-lateralized ERPs, the waveforms at the electrodes C3/4 ipsilateral to the side of the motor response were subtracted from the contralateral ERPs. A jackknife-based scoring method (Miller et al., 1998) was used to determine the onset latencies of the stimulus- and response-locked LRPs. Accordingly, LRP onset latencies were defined as the point in time at which the amplitude reached a pre-defined criterion. As explicitly recommended by Miller et al. (1998), we used 50 and 90% of the maximum amplitude (and adjusted *F*-values accordingly) to determine the stimulus- and response-locked LRP onsets, respectively.

Differences in behavioral [error rates, reaction times (RTs)] and electrophysiological measures (PCN amplitudes/latencies, CDA amplitudes/latencies, sLRP amplitudes/onset latencies, rLRP amplitudes/onset latencies) were analyzed by repeated-measures analyses of variance (ANOVAs) with the factors position sequence (TT, TN, TD) and response sequence (same response, different response). Significant main effects and/or interactions were further examined by means of *post-hoc* comparisons (Tukey HSD). For the sake of brevity, only significant main effects and/or interactions will be reported. Behavioral data were analyzed using “R” (R Core Team, 2012) and Statistica (Version 5). The first three trials in each block (“warm-up” trials), error trials (8%), and trials following an error trial were excluded from the analyses. Finally, trials with RTs slower than 1 s or faster than 200 msec were classified as outliers (2.10%) and not included in the analyses.

## RESULTS

### BEHAVIORAL DATA

#### Error rates

**FIGURE 2:**
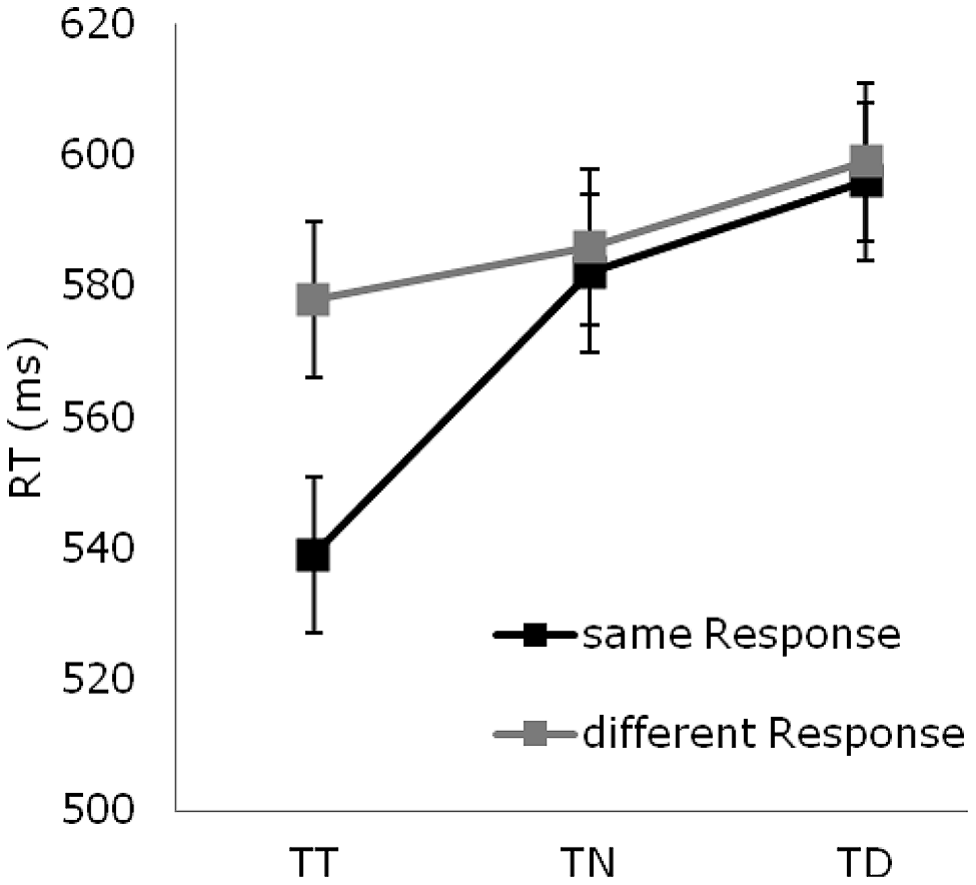
**Reaction time data: mean reaction times (in msec) for targets occurring at previous target (TT), neutral (TN), and distractor (TD) locations, separately for same- (black lines) and different- (gray lines) response trials.** Error bars represent standard error of the mean.

There was a main effect of target position sequence on accuracy [*F*(2,26) = 8.86, *p*< 0.01]: error rates being lowest for targets presented at the previous target location, intermediate for targets at a previously neutral location, and highest for targets at a previous distractor location (7 vs. 8 vs. 9%; all *p* values < 0.05; **Figure 2**). This pattern was more marked for same-response as compared to different-response trials (same response: 5.40 vs. 9.23 vs. 9.21%; different response: 7.79 vs. 7.50 vs. 7.85%), as evidenced by the significant position sequence × response sequence interaction [*F*(2,26) = 9.39, *p*< 0.01].

#### Reaction times

The ANOVA of the RTs revealed all effects to be significant: position sequence [*F*(2,26) = 56.72, *p*< 0.001], response sequence [*F*(1,13) = 8.64, *p*< 0.01], position sequence × response sequence interaction [*F*(2,26) = 35.15, *p*< 0.01]. As can be seen from **Figure 2**, there were RT benefits for targets presented at previous target versus previously neutral locations (558 vs. 584 msec; *p* < 0.001), and RT costs for targets presented at previous distractor versus previously neutral locations (597 vs. 584 msec; *p* < 0.001). The interaction was due to the fact that the benefits for repeated target locations were more marked when participants had to produce the same response, as compared to a different response, as on the previous trial (44 vs. 7 msec; *p* < 0.001). By contrast, the costs for distractor locations were unaffected by whether or not the response was repeated (13 vs. 13 msec)^2^.

### ELECTROPHYSIOLOGICAL DATA

**FIGURE 3:**
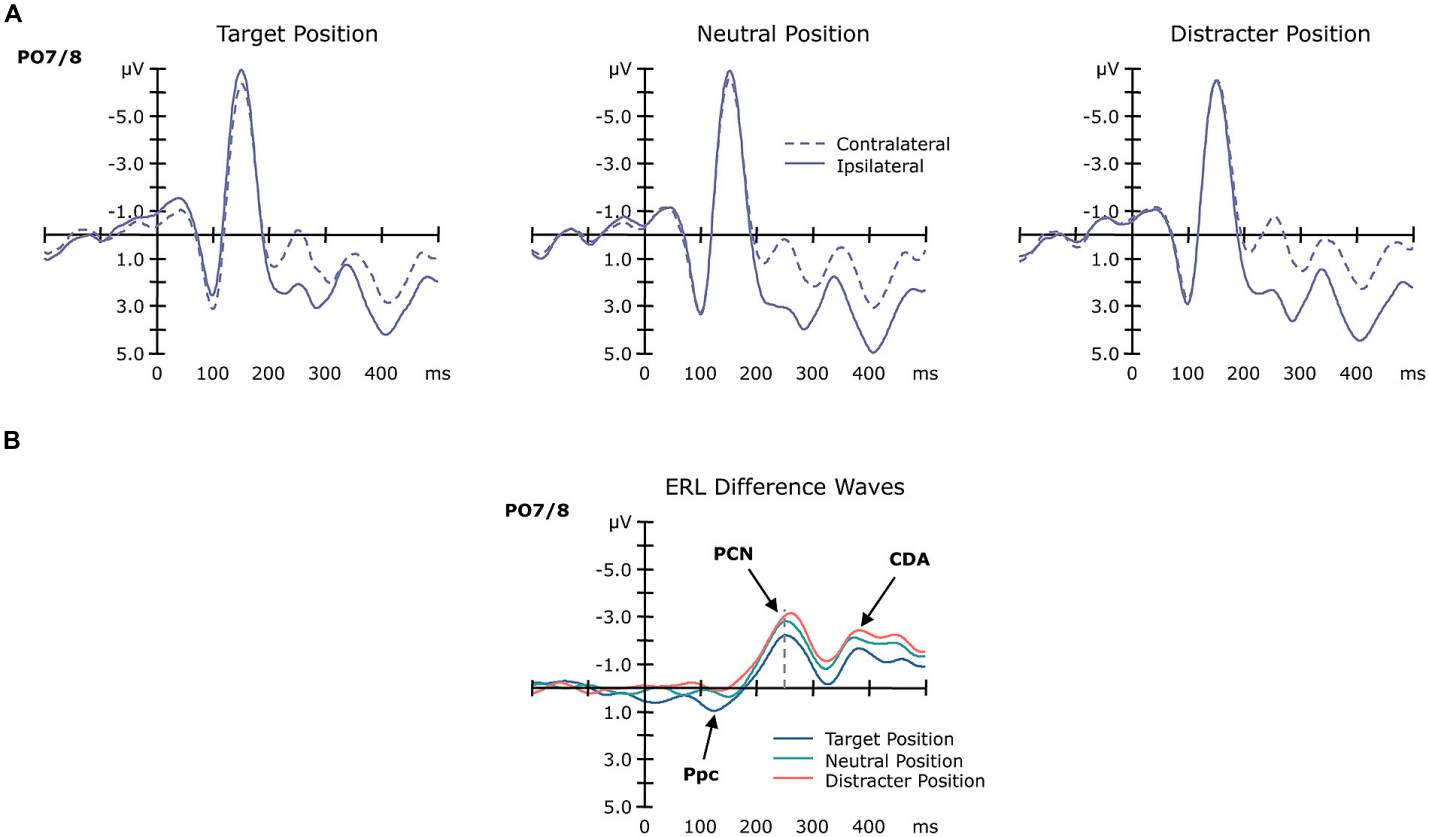
**Grand-average ERP waveforms as a function of target position sequence.** Panel **(A)** shows the ERP waves contralateral (dashed line) and ipsilateral (solid line) to the target position at electrodes PO7/8. Panel **(B)** shows the ERL difference waveforms obtained by subtracting ipsilateral from contralateral activity as a function of the previous target position (blue: target at target, green: target at neutral, red: target at distractor position).

**FIGURE 4:**
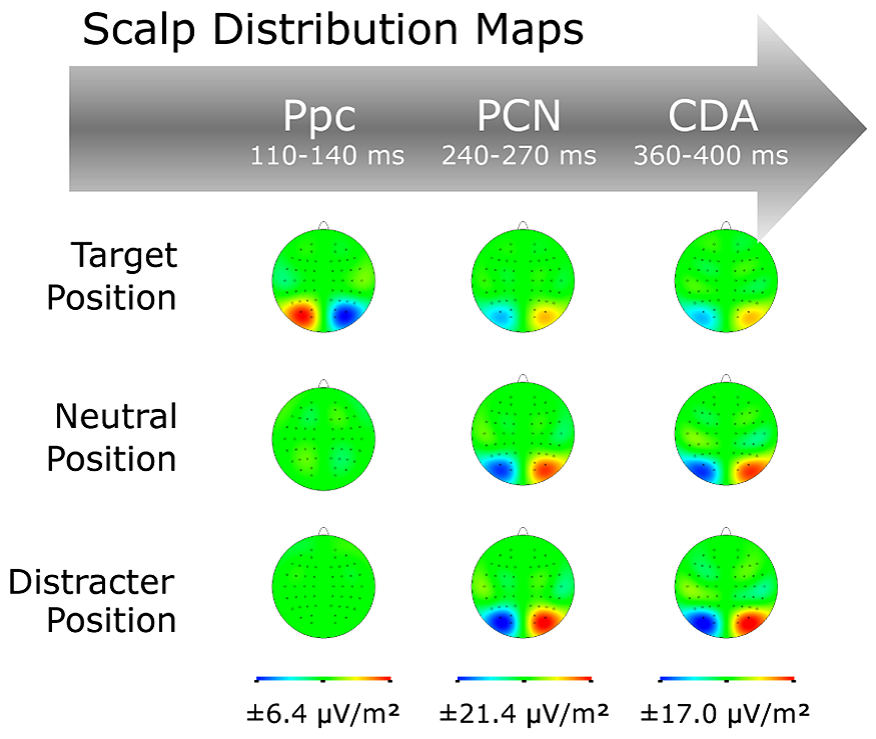
**Scalp distribution maps for the Ppc, PCN, and CDA waves as a function of target position sequence (TT, TN, TD)**.

Grand-average ERP waves contra- and ipsilateral to the target position are illustrated in **Figure 3**, together with the corresponding (contralateral-minus-ipsilateral) difference waves as a function of position sequence (central panel) at electrodes PO7/8, and associated topographical maps (**Figure 4**). As can be seen from these figures, about 200 msec after display onset, there was a negative-going deflection—the PCN wave—that reached its maximum later for targets occurring at previous distractor locations, relative to both targets at previous target and targets at previously neutral locations. Following the PCN, a second negative-going deflection—the CDA wave—can be seen, starting around 350 msec post-stimulus. Both the PCN and CDA appear to be larger in amplitude for targets occurring at previously neutral and, respectively, previous distractor locations, relative to previous target locations. These PCN and CDA amplitude differences might be, however, simply the consequence of an earlier activation difference between the three experimental conditions, in particular: a positive-going deflection between 100 and 150 msec post-stimulus, which is evident exclusively for targets presented at previous target locations. To validate whether this posterior contralateral positivity (Ppc; see also Leblanc et al., 2008; Jannati et al., 2013, for further details) for repeated targets locations was elicited reliably, we additionally analyzed the amplitude of this ERL as function of position sequence and response sequence (see below). As for any other ERL amplitudes in our study, we determined the Ppc amplitudes by averaging five sample points before and after the maximum deflection (within the 50–200-msec time window post-stimulus) for each of the individual experimental conditions.

#### Posterior contralateral positivity (Ppc)

This analysis revealed a significant main effect of position sequence [*F*(2,13) = 18.50, *p*< 0.001], substantiating the pattern evident in **Figures 3** and **4**. *Post-hoc* tests confirmed the Ppc amplitudes to be significantly increased for targets presented at previous target locations (1.28 μV), compared to both targets presented at previously neutral and targets at previous distractor locations (0.68 and 0.33 μV; both *p* values < 0.02).

#### Posterior contralateral negativity (PCN)

For the PCN, the main effect of target position reached significance for both the component’s amplitude [*F*(2,13) = 13.45, *p* < 0.001] and its timing [*F*(2,13) = 3.91, *p* < 0.05]. PCN amplitudes were numerically lowest for targets at previous target locations (-2.32 μV), intermediate for targets at neutral locations (-2.99 μV), and highest for targets at distractor locations (-3.24 μV). *Post-hoc* tests confirmed the amplitude difference between the previous target and distractor locations (*p* = 0.02). For the PCN timing, we found latencies being shortest for targets at previous target locations (256 msec), intermediate for targets at neutral locations (261 msec), and slowest for targets at distractor locations (275 msec). *Post-hoc* tests revealed the PCN latency to be significantly slower for targets at previous distractor locations compared to both targets at neutral and targets at target locations (both *p* values < 0.05). Note that there was no effect involving the factor response sequence, neither on the PCN amplitude nor its latency (accordingly, the ERLs in **Figure 3B** are collapsed across same- and different-response trials, to illustrate their sensory-driven nature).

#### Contralateral delay activity (CDA)

For the CDA, the main effect of target position sequence was revealed significant for the CDA amplitudes [*F*(2,13) = 18.57, *p* < 0.001]. As confirmed by *post-hoc* tests, CDA amplitudes were more pronounced for targets at previously neutral and previous distractor locations relative to targets at previous target locations (-2.82 and -2.63 μV vs. -1.91 μV; both *p* values < 0.05).

#### Stimulus-locked lateralized readiness potential (sLRP)

**FIGURE 5:**
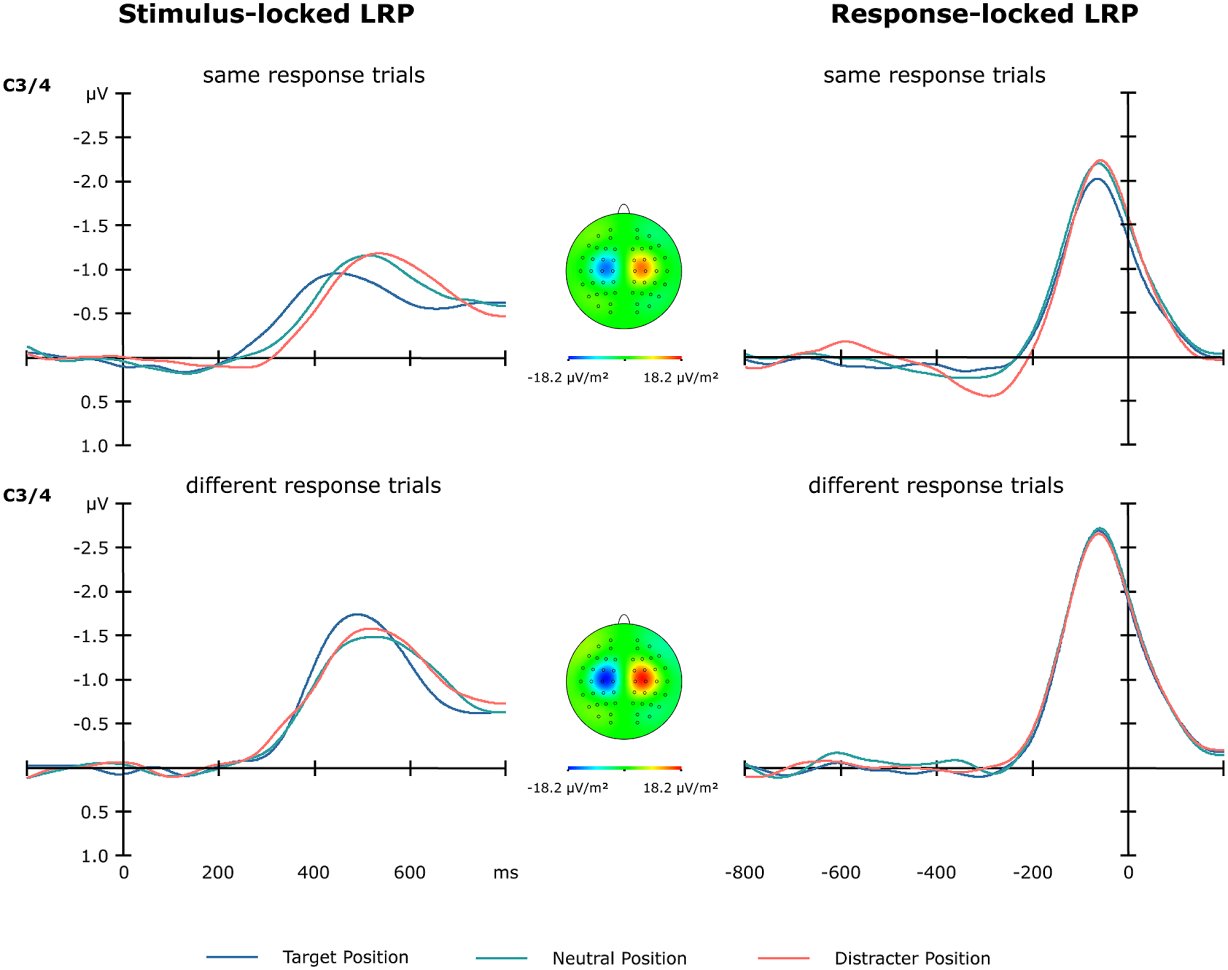
**Lateralized readiness potentials obtained from electrodes C3/4 as a function of target position sequence (blue line: target at previous target location, green line: at previous neutral location, red line: at previous distractor location) and motor-response sequence (same vs. different response as on the previous trial).** The left panel shows the stimulus-locked LRPs in the 800-msec time window following stimulus onset. The right panel shows the response-locked LRPs in the 800-msec time window prior to response onset.

As can be seen from **Figure 5**, cross-trial response repetitions versus changes had a significant effect on the sLRP amplitude [*F*(1,13) = 15.53, *p* < 0.002], with different-response trials exhibiting stronger amplitudes (-1.80 μV) than same-response trials (-1.31 μV). For sLRP onset latencies, the main effect of position sequence [*F*(2,13) = 8.42, *p* < 0.05] and the position sequence × response sequence interaction [*F*(1,13) = 5.20, *p* < 0.05] were significant. For same-response trials, the sLRP onset latencies were shorter for targets presented at previous target locations compared to targets at previously neutral and targets at previous distractor locations (325 vs. 389 and 410 msec; both *p* values < 0.05). By contrast, there were no reliable position-dependent differences for different-response trials (376, 377, and 382 msec).

#### Response-locked lateralized readiness potential (rLRP)

As expected from the sLRP analyses, cross-trial repetition versus change of the motor response modulated the amplitude of the rLRP [*F*(1,13) = 17.47, *p* < 0.001]: rLRP amplitudes were increased on different-response relative to same-response trials (-2.80 vs. -2.27 μV; **Figure 5**). The rLRP onset latency, by contrast, was not affected by position or response sequences.

## DISCUSSION

The aim of the present study was to explore the neural mechanisms underlying pPoP effects—target facilitation and distractor inhibition—in visual search. By examining RT performance in combination with specific sensory- and motor-response-related ERLs, the time course of four distinct levels of visual processing could be dissociated. The behavioral effects replicated previous findings (e.g., Maljkovic and Nakayama, 1996; Gokce et al., 2013) of responses being expedited to targets presented at previous target locations and slowed to targets at previous distractor locations, relative to a neutral (baseline) condition with targets appearing at previously empty locations. This pattern is indicative of processing being facilitated when, across trials, the target is again presented at the previous target location and inhibited when it appears at one of the previous distractor locations. What follows is a discussion of the space- and response-related modulations of the analyzed ERLs that accompany these RT effects.

### NO PRIMING OF POP-OUT FOR REPEATED TARGET LOCATIONS

The most striking finding was exhibited by the PCN wave—a well established and generally agreed ERP marker of focal-attentional selection of task-relevant target items (e.g., Eimer, 1996; Woodman and Luck, 1999; Töllner et al., 2012a)—which was significantly slowed for targets presented at previous distractor locations relative to both targets presented at previous neutral and targets at previous target locations^3^. Combined with the RT effects, this pattern indicates that distractor location inhibition affects (i.e., prolongs) the time required to select the target by focal attention, whereas target location facilitation modulates processing primarily, if not exclusively, after focal-attentional selection. With regard to PCN magnitude, the reduced amplitude for targets presented at previous target locations relative to both the target-at-neutral and target-at-distractor location conditions suggests, at first glance, that the overall salience computed for the target is reduced when it appears at the same location on consecutive trials. This would be closely in line with previous studies (Conci et al., 2011; Töllner et al., 2011) in which the PCN was likewise reduced for lower relative to higher target salience (see also McDonald et al., 2009, for reduced PCN waves when target locations were repeated within the time frame of IOR). With regard to the present study, however, such an interpretation has to remain speculative, as the PCN amplitude difference—and any difference in the subsequent ERL waves—may simply reflect a cascaded activation difference originating, e.g., from the preceding Ppc^4^.

Of theoretical importance, the Ppc wave was elicited selectively by targets occurring at the same location as on the previous trial. A recent study by Jannati et al. (2013) suggested that this early sensory ERL may represent the physical distinctiveness of an item relative to its neighbors, independently of whether this item is a target or a distractor singleton. Note, however, that Jannati et al. (2013) used an additional-singleton paradigm, in which a task-irrelevant distractor singleton could co-occur either within the same or the opposite hemifield as the target singleton. Using this design, these authors found a positivity posterior contralateral to the salient color singleton, regardless of whether that singleton was an attended target or an ignored distractor. Applied to the present data, however, this saliency-based notion would imply that targets generate a strong bottom-up (i.e., pop-out) signal only when they occur repeatedly at the same location. This would appear rather unlikely, however, because if the Ppc indeed reflects physical item conspicuity, a Ppc should have also been evident in the present target-at-neutral and target-at-distractor-location conditions, in which the target was singled out from the distractors by the very same, salient color feature difference.

Hence, the Ppc triggered in the present study may instead reflect a different mechanism: some type of location-specific inhibition that tags target positions for subsequent trial episodes, suppressing early sensory coding when the target occurs at exactly the same position as on the previous trial. Such an inhibitory mechanism would also be in line with the lack of a PCN timing advantage for this particular condition, and it would fit with observations that have linked location/hemifield-specific inhibition to increased power in the alpha band (e.g., Sauseng et al., 2005; Klimesch et al., 2006). Sauseng et al. (2005), for instance, used a spatial cueing task, in which an arrow indicated the to-be-attended (left vs. right) visual hemifield at the start of each trial. Participants had to covertly attend to the cued hemifield (75% cue validity), and discriminate the size of a target stimulus (small vs. large). In the time period following the cue, Sauseng et al. (2005) found significantly stronger alpha amplitudes at parieto-occipital electrodes ipsilateral to the cued hemifield, that is, contralateral to the non-cued (i.e., “suppressed”) hemifield. Based on this and a number of follow-up studies (see Klimesch et al., 2006, for a review), this increase in alpha power was taken to reflect a general inhibitory mechanism. Thus, conceivably, the present Ppc may reflect such an event-related synchronization in the alpha band and, thus, the associated inhibitory mechanism (for repeated target locations). However, whether there is indeed such a link remains an open issue to be addressed in future studies.

### REDUCED RECURRENT PROCESSING DEMANDS AT THE PREVIOUS TARGET LOCATIONS?

Following the PCN, the CDA wave was likewise reduced in amplitude when the target was presented at a previous target location, compared to both neutral and distractor locations. While the CDA has originally been observed in WM tasks (e.g., Vogel and Machizawa, 2004; Reinhart and Woodman, 2013), a number of recent studies have identified this ERL also in visual-search paradigms (e.g., Mazza et al., 2007; Töllner et al., 2013; Wiegand et al., 2013a), provided that the task required precise target identification. In more detail, while the CDA amplitude is primarily determined by individuals’ visual short-term memory storage capacity (parameter “k”; e.g., Vogel and Machizawa, 2004; Wiegand et al., 2013b), another factor influencing the CDA signal is the difficulty associated with retrieving task-critical visual information maintained in WM (Töllner et al., 2013). In the study of Töllner et al. (2013), participants had to perform a compound-search task under conditions in which the visual search displays were blurred to varying degrees (by applying different Gaussian kernels)—selectively impacting the precise orientation information that was required to select the correct response (vertical vs. horizontal), but not the color information that singled out the target from amongst the distractors (red vs. green). This manipulation affected the signal strength of the CDA (but not the PCN) wave, with CDA amplitudes increasing gradually with decreasing stimulus contrast. This finding demonstrates that, in visual attention tasks, CDA amplitudes might further index the amount of post-selective recurrent feedback processing recruited to extract detailed object information from WM.

Thus, with regard to the present findings, the CDA effect obtained may be taken to indicate that location-specific post-selective feedback connections may be primed across trials when the target re-occurs at the same position. Restated, for repeated target locations, positional priming modulates target processing only after it has been selected, such that participants have to engage in less recurrent processing to reliably identify the target’s orientation in WM. However, as already stated above, this suggestion must remain speculative, as any amplitude differences during the CDA time period may simply be a consequence of the earlier activation differences in the ERL waves.

### SPACE- AND RESPONSE-BASED INTERTRIAL DYNAMICS DETERMINE RESPONSE DECISIONS INTERACTIVELY

Mirroring the RT pattern, the present findings revealed the time required to decide upon the appropriate motor response to interactively depend on both position sequence and response sequence: relative to the neutral baseline, the sLRP timing was overall faster for targets at previous target locations, and slower for targets at previous distractor locations. Of note, for repeated target locations, the RT benefit was even more pronounced when participants had to produce the same motor response as on the previous trial. Since there was no such interaction between target sequence and response sequence, this interaction must occur at an intermediate stage of processing—after target selection but before response production—in which the response is selected in accordance with a pre-established S–R rule. One putative mechanism that can account for this interactive pattern has been referred to as “combined expectancies” (see Kingstone, 1992; Töllner et al., 2008) – the idea being that the central (i.e., response selection) processing system implicitly assumes a cross-trial coupling of two (or more) stimulus attributes that relate to one-and-the-same object (or processing episode), even though the attributes are statistically uncorrelated. That is, when a primary target attribute (e.g., its position: “left”) is repeated/changed across trials, the system assumes that other stimulus attributes (the response-defining feature: “top-notch,” or the associated response itself) will be repeated/changed, too, thus shortening processing times when these attributes are actually repeated/changed. However, if only one of the two stimulus attributes repeats/changes, these expectancies are violated and central-stage processing would need to start from scratch, resulting in a processing time cost. Note that exactly this pattern was revealed by the present data (see **Figure 5**): for same-response trials, the sLRP onset occurred earlier—over and above the PCN advantage—for repeated as compared to changed target positions. By contrast, the sLRP latencies were statistically equivalent for all different-response conditions, implying that the earlier PCN advantage evident for repeated target locations was abolished at the response selection stage. Thus, these findings provide additional ERL evidence for the “combined-expectancies” notion originally proposed by Kingstone (1992), extending this pattern from non-spatial stimulus attributes (i.e., target-defining dimensions: Töllner et al., 2008; and sensory modalities: Töllner et al., 2012c) to spatial stimulus attributes (i.e., target locations).

### MOTOR PROCESSING IS INDEPENDENT OF SPACE-BASED INTERTRIAL DYNAMICS

Finally, the present findings revealed stronger response-locked LRPs for cross-trial changes versus repetitions of the motor response, with no further modulations by the previous placement of the target. This response sequence-specific pattern replicates previous studies (Töllner et al., 2008, 2010), which led to the proposal of a “response-weighting” account to explain the boosted rLRP signals. In detail, similar to visual dimensions, the processing of a given motor response may implicitly leave a response-specific memory trace in the motor system that biases the re-activation of the identical effectors across trials. In other words, newly activated responses may require the accumulation of more relative to less neural evidence—as reflected by the enhanced rLRP waves—to reach a response-initiating threshold in the motor system. Critically, this response-weighting mechanism operates independently of the non-spatial (Töllner et al., 2008) and spatial (present study) intertrial dynamics.

## CONCLUSION

In conclusion, in their pioneering study on pPoP, Maljkovic and Nakayama (1996) reported RT benefits and costs for targets at previous target and distractor locations, respectively, relative to “neutral” locations. The results of the current ERL study replicates this RT pattern and demonstrates that these two positional priming effects—target (location) facilitation and distractor inhibition—are indeed independent phenomena, originating from distinct stages in the visual processing system. The most important conclusion is that, at variance with Maljkovic and Nakayama’s (1996) original proposal, presenting the target at the same position as on the previous trial does not yield a stronger pop-out effect, that is, shortened processing at (or prior to) the stage of visual selection. Instead, target location facilitation arises from expedited processing only after focal-attentional target selection, such as response selection (and, presumably, recurrent target identification) processes. By contrast, and in line with Maljkovic and Nakayama (1996), distractor location inhibition affects pre-selective target coding stages.

## AUTHOR CONTRIBUTIONS

Ahu Gokce, Thomas Geyer, Kathrin Finke, Hermann J. Müller, and Thomas Töllner designed research; Ahu Gokce performed data acquisition; Ahu Gokce, Thomas Geyer, and Thomas Töllner analyzed and interpreted the data; and Ahu Gokce, Thomas Geyer, Hermann J. Müller, and Thomas Töllner wrote the paper.

## Conflict of Interest Statement

The authors declare that the research was conducted in the absence of any commercial or financial relationships that could be construed as a potential conflict of interest.

## Footnotes

1 In their Experiment 2, Eimer et al. (2010) further showed that performance gains in the repeated condition were due to both repetition of the target and repetition of the distractor color (for evidence in favor of distinct target and distractor feature priming, see also Lamy et al., 2008; Kristjánsson and Driver, 2008, as well as Burra and Kerzel, 2013, who however used a different paradigm).

2 Maljkovic and Nakayama (1996) found a gradient of facilitation around previous target locations. To test whether facilitation is graded in the current study, we re-analyzed the facilitatory effect (i.e., the RT difference between the target-at-target- and target-at-neutral-location conditions) as a function of the spatial separation between the previous and the current target position. For example, if the target on trial *n-1* appeared at the top-left position in a “square” configuration and on trial *n* at the left (right) position in a “diamond” configuration, this transition was referred to as *near* (*far*) target location distance condition. A gradient of facilitation centered on the target position on trial *n-1* would encompass near locations, thus expediting RTs to targets at then near *neutral* locations on trial *n* and thus rendering the facilitatory effect, estimated by subtracting RTs in target-at-target location from RT in the *near* target-at-neutral location condition, smaller. The results of the additional analysis confirmed this prediction: facilitatory priming was reliably reduced for near relative to far cross-trial target location distances (20 vs. 32 ms; *p* = 0.01), suggesting that facilitation was spatially graded in the present study. The re-analysis of facilitatory priming as a function of cross-trial target location distance is interesting in another respect. Previous research has shown that facilitatory priming is influenced by repetitions vs. changes of the overall “Gestalt” configuration (here: diamond vs. square configuration; see Gokce et al., 2013—the core finding being that facilitation for target locations was larger in same- relative to different-configuration trials). Given this, the distance analysis provides a “pure” measure of the effect of repeated target location (and its variation as a function of the cross-trial target distance), unconfounded by the effects of repeated vs. changed configurations. This is because both the near and far target location distance condition were accompanied by a change of the overall configuration across trials.

3 It should be noted that it is hazardous to interpret PCN timing effects when there may be temporally overlapping components and/or amplitude differences. In the present case, however, we believe that our proposal of delayed selection in the “distractor location” condition is tenable, for the following three reasons: First, the “target-at-neutral-location” condition was statistically comparable (i.e., there were no statistical differences relative) to the “target-at-distractor-location” condition during the time windows of the Ppc, PCN, and the subsequent positive-going deflection. Thus, the PCN latency shift between neutral and distractor location trials cannot be attributed to temporally overlapping components and/or amplitude differences. Second, the comparable width of the PCN waves: To further examine for a putative impact of preceding/following positive potentials, or variance differences, on the PCN, we conducted a repeated-measure ANOVA analyzing PCN width (defined by subtracting PCN onsets from PCN offsets at 50% of the maximum amplitude, respectively) as a function of target location sequence. A significant impact of putative preceding/following positive potentials on the PCN should have resulted in reduced PCN widths in the affected conditions. However, this effect was far from statistical significance (*p* > 0.72), effectively ruling out this alternative explanation. Third, the symmetric shape of the three PCN difference waves: Again, if there had been a critical modulation of the preceding and/or following positivity, one would have expected a rather asymmetric PCN shape (with a somewhat steeper ascending part for the target location condition, and/or a somewhat sharper descending part for the distractor location condition), which was, however, not the case (see **Figure 3**).

4 As can be seen in **Figure 3B**, the conditions seem to differ already before the onset of the Ppc, which may suggest that the current ERL amplitude differences may be associated with a slow drift rather than the cascaded activation difference first measureable in the Ppc (we thank an anonymous reviewer for pointing to this alternative interpretation).
